# Establishment of the Myeloid TBX-Code Reveals Aberrant Expression of T-Box Gene TBX1 in Chronic Myeloid Leukemia

**DOI:** 10.3390/ijms25010032

**Published:** 2023-12-19

**Authors:** Stefan Nagel, Josephine Haake, Claudia Pommerenke, Corinna Meyer, Roderick A. F. MacLeod

**Affiliations:** Leibniz-Institute DSMZ, 38124 Braunschweig, Germany

**Keywords:** AML, dasatinib, ETS-code, homeobox, imatinib, NKL-code, TALE-code

## Abstract

T-box genes encode transcription factors, which control developmental processes and promote cancer if deregulated. Recently, we described the lymphoid TBX-code, which collates T-box gene activities in normal lymphopoiesis, enabling identification of members deregulated in lymphoid malignancies. Here, we have extended this analysis to cover myelopoiesis, compiling the myeloid TBX-code and, thus, highlighting which of these genes might be deregulated in myeloid tumor types. We analyzed public T-box gene expression datasets bioinformatically for normal and malignant cells. Candidate T-box-gene-expressing model cell lines were identified and examined by RQ-PCR, Western Blotting, genomic profiling, and siRNA-mediated knockdown combined with RNA-seq analysis and live-cell imaging. The established myeloid TBX-code comprised 10 T-box genes, including progenitor-cell-restricted TBX1. Accordingly, we detected aberrant expression of TBX1 in 10% of stem/progenitor-cell-derived chronic myeloid leukemia (CML) patients. The classic CML cell line K-562 expressed TBX1 at high levels and served as a model to identify TBX1 activators, including transcription factor GATA1 and genomic amplification of the TBX1 locus at 22q11; inhibitors, including BCR::ABL1 fusion and downregulated GNAI2, as well as BMP, FGF2, and WNT signaling; and the target genes CDKN1A, MIR17HG, NAV1, and TMEM38A. The establishment of the myeloid TBX-code permitted identification of aberrant TBX1 expression in subsets of CML patients and cell lines. TBX1 forms an integral part of an oncogenic regulatory network impacting proliferation, survival, and differentiation. Thus, the data spotlight novel diagnostic markers and potential therapeutic targets for this malignancy.

## 1. Introduction

Myelopoiesis starts with hematopoietic stem cell (HSC)-derived common myeloid progenitors (CMPs) in the bone marrow, finally generating several types of immune and blood cells, including granulocytes, monocytes, dendritic cells, and erythrocytes. Developmental processes in the hematopoietic compartment are mainly regulated at the transcriptional level [1,2]. Accordingly, specific transcription factors (TFs) control differentiation into mature immune cells along the myeloid lineage, for example, CEBPA, GATA1, GATA2, HOXA9, RUNX1/AML1, and SPI1/PU.1 [3,4,5,6,7]. Deregulation of these TFs via chromosomal rearrangements or gene mutations generates myeloid malignancies, highlighting their pathogenic significance [3,4,5,6,7]. Therefore, investigation of developmental TFs is likely to promote understanding of both normal myelopoiesis and myeloid tumorigenesis.

The human genome encodes about 1600 TFs, which are classified systematically according to similarities in sequence and structure [8]. Two main groups of TFs are characterized by the presence of a particular DNA-binding domain, namely, the homeodomain and the T-box domain, respectively. Both TF groups control basic developmental processes and their mutation or deregulation causes developmental diseases or cancer [9,10,11,12]. The homeodomain forms three alpha-helices separated by two loops, characteristics of the helix–turn–helix family of TFs. It consists of 60 amino acid residues and Helix 3 interacts in a sequence-specific manner with the major groove of DNA [13]. Homeobox genes have been arranged into eleven classes and several subclasses according to similarities in their sequences [14]. In contrast, the T-box domain is three-fold longer than the homeodomain and consists of some 180 amino acid residues. It forms a seven-stranded beta-barrel structure, which belongs to the s-type immunoglobulin domain class [15]. T-box factors interact with the major and minor grooves of DNA as monomers or dimers [15,16]. Thus, homeodomain and T-box TFs are distinguished by the sequence and structure of their DNA-binding domains while sharing basic regulatory impacts in developmental processes.

TF gene codes describe normal expression patterns of TF subgroups at particular developmental stages, tissues, or compartments. They allow the detection and evaluation of deregulated TFs in corresponding tumor types, which might prove useful for diagnostics and, eventually, therapeutics [10,17]. We have already established the NKL-code, which shows the activity of NKL subclass homeobox genes in the course of blood and immune cell development covering early hematopoiesis, lymphopoiesis, and myelopoiesis [10]. In addition, we have described the TALE-, TBX-, and the ETS-codes, displaying physiological signatures of respective TALE class homeobox genes, T-box genes, and ETS genes in lymphopoiesis [10,18,19,20]. We have exploited these paradigms to detect and validate transcriptional irregularities denoting oncogenic activities for particular NKL, TBX, and ETS genes [10,18,19,20].

Here, we followed an analogous approach to study T-box gene expression in early hematopoiesis and during myelopoiesis [21,22]. The human genome contains seventeen T-box genes, which are arranged in five subfamilies, namely, T (containing TBXT and TBX19); Tbx1 (TBX1, TBX10, TBX15, TBX18, TBX20, TBX22); Tbx2 (TBX2, TBX3, TBX4, TBX5); Tbx6 (TBX6, MGA); and Tbr1 (EOMES, TBX21, TBR1). This gene classification neatly recaptures their evolutionary history in metazoa [11]. While a few members have been examined in more detail in immune cells, including TBX21 (alias Tbet) in B-cells, NK-cells, T-cells, and innate lymphoid cells (ILCs) [23,24,25,26] and EOMES in T-cells, NK-cells, and ILC1 [26,27,28,29,30,31], the developmental role and expression of remaining the T-box genes in hematopoiesis is largely unknown.

Recently, we reported on the physiological activities of T-box genes in lymphopoiesis, contributing to the understanding of this group of TFs in B-cell development and enabling identification of aberrantly expressed TBX3 in Hodgkin lymphoma [19]. Our current investigations presented here reveal 10 T-box genes, specifically expressed in early hematopoiesis and myelopoiesis. We termed the resultant physiological expression pattern “myeloid TBX-code”, showing their role in the differentiation of myeloid immune cells. Furthermore, we exploited this TBX-code to identify aberrantly expressed TBX1 in patients of chronic myeloid leukemia (CML). CML is a stem-cell-derived cancer containing the fusion gene BCR::ABL1. Fusion partner ABL1 encodes a tyrosine kinase, which drives hematopoietic tumorigenesis if aberrantly overexpressed and activated. The design of ABL1 inhibitors, like imatinib or dasatinib, represented milestones for the therapy of CML. However, the emergence of resistance to these drugs underlines the requirement of alternative therapeutic targets and approaches [32,33,34,35].

## 2. Results

### 2.1. Myeloid TBX-Code

**Figure 1:**
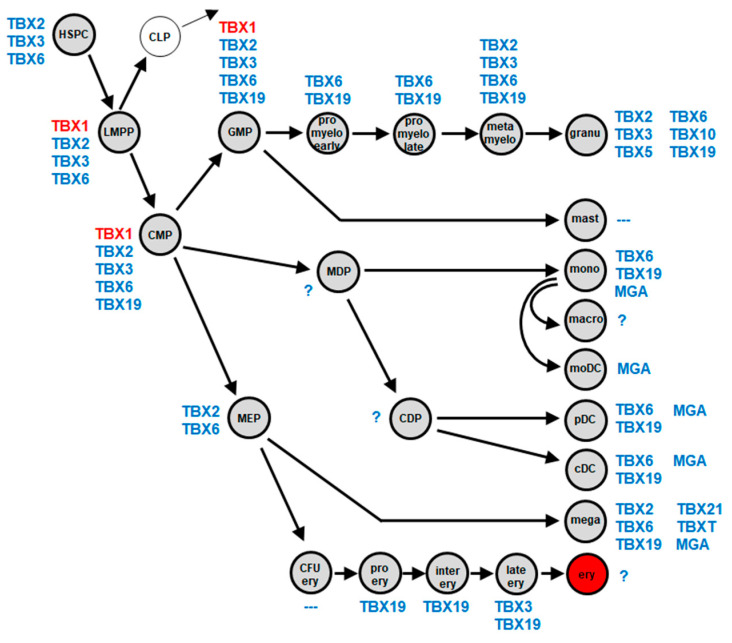
The myeloid TBX-code summarizes T-box gene activities (blue, TBX1 in red) in hematopoietic stem and progenitor cells, as well as in mature myeloid immune and blood cells. Question marks show entities lacking information about gene activities. Abbreviations: cDC, conventional dendritic cell; CDP, common dendritic progenitor; CFU ery, erythroid colony forming unit; CLP, common lymphoid progenitor; CMP, common myeloid progenitor; ery, erythrocyte; GMP, granulo-myeloid progenitor; granu, granulocyte; HSPC, hematopoietic stem and progenitor cell; inter ery, intermediate-stage erythroblast; late ery, pyknotic-stage erythroblast; LMPP, lymphoid and myeloid primed progenitor; macro, macrophage; mast, mast cell; MDP, monocyte dendritic cell progenitor; mega, megakaryocyte; MEP, megakaryocytic-erythroid progenitor; metamyelo, metamyelocyte; moDC, monocyte-derived dendritic cell; mono, monocyte; pDC, plasmacytoid dendritic cell; pro ery, pro-erythroblast; pro myelo early/late, early/late promyelocyte.

To reveal physiological activities of T-box factors in early hematopoiesis and myelopoiesis, we analyzed, in accordance with our previous studies, public gene expression profiling and RNA-seq datasets [21,22]. The resultant expression data for all 17 T-box genes are provided in Figures S1–S5. The generated gene signature has been named “myeloid TBX-code” and is depicted in Figure 1. This code covers 10 T-box genes: MGA, TBX1, TBX2, TBX3, TBX5, TBX6, TBX10, TBX19, TBX21, and TBXT. MGA was restricted to mature cells, including monocytes, dendritic cells (DCs), and megakaryocytes. In contrast, TBX1 expression was only found in progenitor/stem cells, including the lymphoid and myeloid primed progenitor (LMPP), granulo-myeloid progenitor (GMP), and common myeloid progenitor (CMP). TBX6 was expressed in all entities except mast cells, monocyte-derived DCs, and the erythropoietic lineage while TBX10 expression was restricted to mature granulocytes. TBX19 was expressed in all entities except mast cells and monocyte-derived DCs while TBX21 and TBXT were only expressed in megakaryocytes.

Thus, T-box genes show a specific expression pattern in entities of developing and mature myeloid cells, which supports their reported role in differentiation processes. Deregulation of specific T-box genes may, therefore, disturb myeloid development and support leukemogenesis.

### 2.2. Expression of TBX1 in Myeloid Malignancies

To study T-box gene deregulation in myeloid malignancies, we examined their expression in acute myeloid leukemia (AML) and chronic myeloid leukemia (CML) patients using public expression profiling data. We analyzed dataset GSE15434 covering 251 AML patients with normal karyotypes (Figure S6), dataset GSE14468 containing 526 AML patients with diverse aberrations and mutations (Figure S7), and dataset GSE44589 containing 198 CML patients prior to and after treatment with imatinib (Figure S8). Collectively, the data showed aberrant expression of EOMES, MGA, TBX1, TBX2, TBX3, TBX5, TBX6, TBX10, TBX15, TBX19, TBX21, and TBR1 in AML and of EOMES, TBX1, TBX2, TBX4, TBX19, and TBX21 in CML. This high incidence of aberrantly activated T-box genes in AML and CML may well reflect the fundamental role of this group of TFs in myeloid development.

In the following, we focused on deregulated TBX1, which showed normal activities in stem/progenitor cells and may, thus, support stemness and inhibit myeloid differentiation, representing an oncogenic hallmark. TBX1 was found to be aberrantly expressed in 3% of AML patients with normal karyotypes and 10% of CML patients prior to treatment with imatinib. CML is derived from stem/progenitor cells, which may underlie a higher predisposition and incidence of aberrant TBX1 activation in this type of myeloid malignancy.

**Figure 2:**
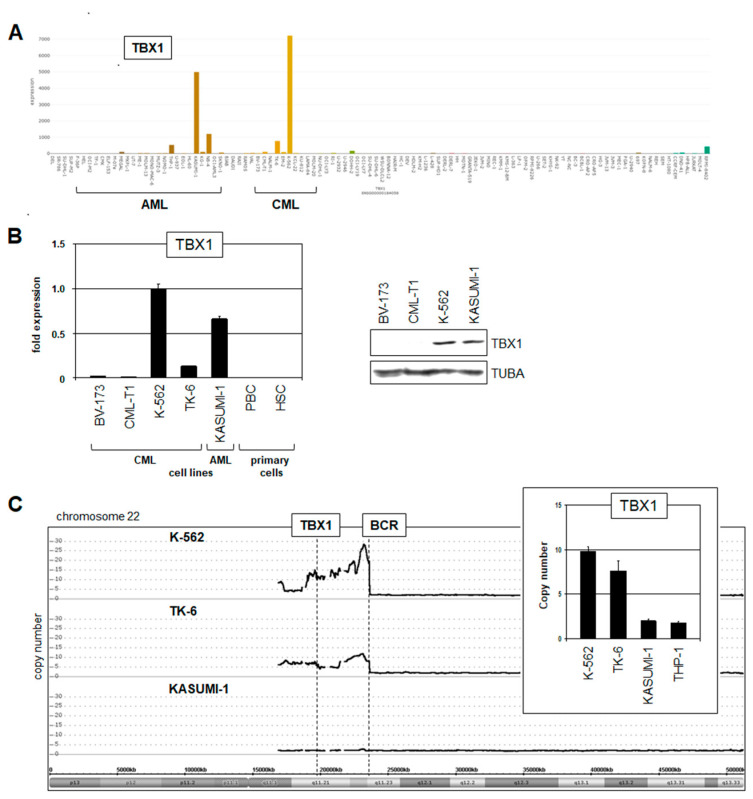
TBX1 expression and genomic copies in myeloid tumor cell lines. (**A**) Analysis of the RNA-seq dataset LL-100 showed aberrant activation of TBX1 in cell lines derived from AML (KASUMI-1, NB-4, THP-1) and CML (K-562, TK-6). (**B**) RQ-PCR (left) and Western Blot analysis (right) demonstrated elevated TBX1 levels in K-562 and KASUMI-1. According to the TBX-code, hematopoietic stem cells (HSCs) and peripheral mononuclear blood cells (PBCs) did not express TBX1 (left). (**C**) Genomic profiling data for Chromosome 22 revealed amplification of the TBX1 and BCR loci in CML cell lines K-562 and TK-6, contrasting with AML cell line KASUMI-1. These copy number alterations were confirmed by genomic RQ-PCR analysis, setting KASUMI-1 at diploidy (insert).

To search for TBX1-expressing model cell lines, we screened RNA-seq dataset LL-100, comprising a representative panel of 100 leukemia/lymphoma cell lines [36]. Accordingly, TBX1 was significantly expressed in AML cell lines KASUMI-1, NB-4, and THP-1 and in CML cell lines K-562 and TK-6 (Figure 2A). Interestingly, no significant TBX1 activity was detected in cell lines derived from lymphoid malignancies, highlighting its oncogenic role in the myeloid compartment. RQ-PCR analysis confirmed high TBX1 expression levels in K-562 and KASUMI-1 and Western Blot analysis demonstrated its expression at the protein level in both cell lines (Figure 2B), endorsing their candidacy to serve as TBX1 models in myeloid malignancies.

Chromosomal rearrangements and copy number alterations may underlie aberrant expression of oncogenes, concurrently spotlighting the pathological significance of their activation. However, analysis of reported karyotypes from AML cell lines KASUMI-1, NB-4, and THP-1 discounted rearrangements at TBX1 located at chromosomal position 22q11.21. In contrast, CML cell lines K-562 and TK-6 are BCR::ABL1-positive and, thus, bear 22q11.23 alterations, which are cytogenetically indistinguishable from potential TBX1 rearrangements. However, copy number analysis demonstrated the co-amplification of TBX1 and BCR in K-562 and TK-6 while KASUMI-1 and THP-1 maintained wild-type configurations (Figure 2C).

Taken together, TBX1 is aberrantly activated in subsets of AML and CML patients and cell lines. TBX1 overexpression correlated with genomic amplifications at 22q11 in the CML cell lines K-562 and TK-6, indicating a pathogenic connection to the hallmark fusion gene BCR::ABL1 and uncovering the oncogenic role of TBX1 in CML. Therefore, we focused our study on deregulated TBX1 in CML.

### 2.3. Deregulation of TBX1 in CML

**Figure 3:**
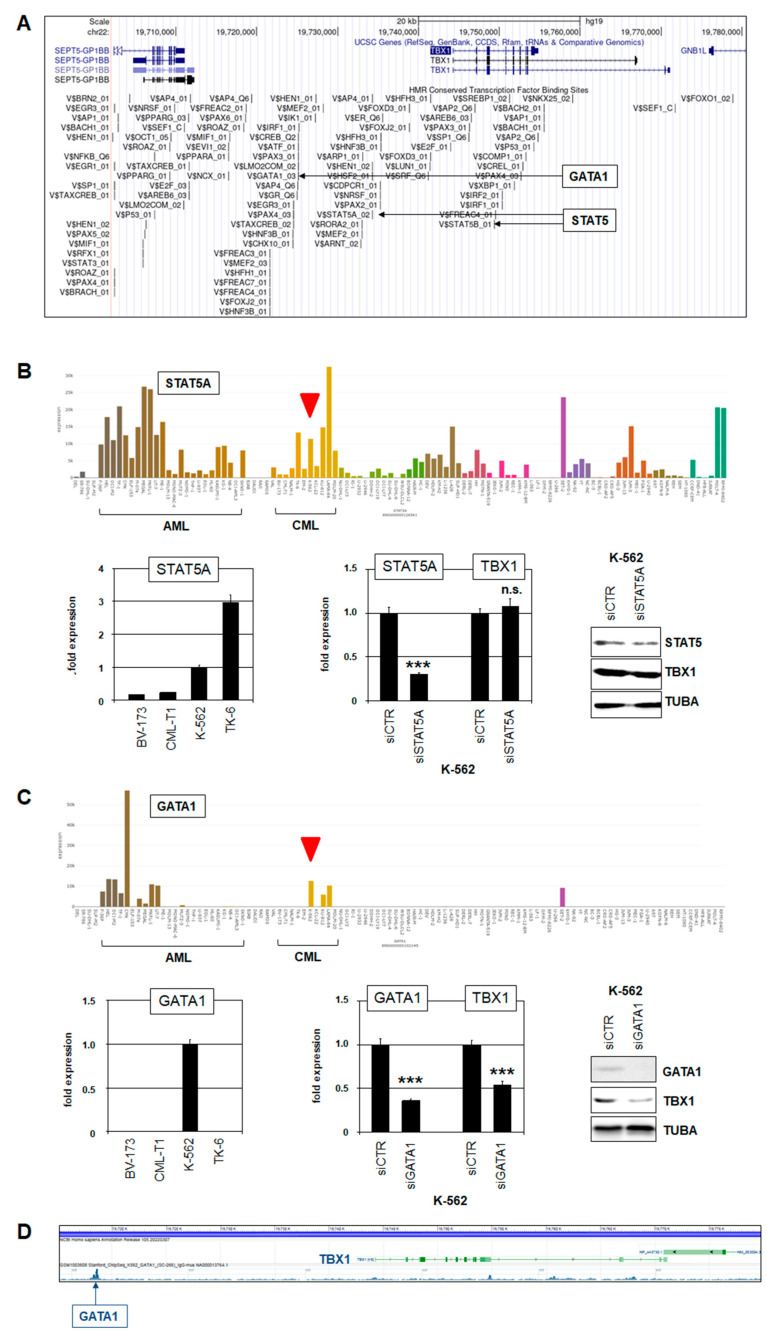
GATA1 activates TBX1 in CML cell line K-562. (**A**) Potential TF binding sites at TBX1 were obtained from the UCSC genome browser. Binding sites for GATA1 and STAT5 are highlighted. (**B**) RNA-seq expression data for STAT5A in 100 leukemia/lymphoma cell lines showing elevated levels in K-562 (red arrowhead). AML and CML cell lines are indicated (above). RQ-PCR analysis of STAT5A in CML cell lines confirmed elevated STAT5A expression in K-562. siRNA-mediated knockdown of STAT5A in K-562 cells spared TBX1 expression. Western Blot analysis showed STAT5A reduction at the protein level after knockdown (below). (**C**) RNA-seq expression data for GATA1 in 100 leukemia/lymphoma cell lines showing elevated levels in K-562 (red arrowhead). AML and CML cell lines are indicated (above). RQ-PCR analysis of GATA1 in CML cell lines confirmed elevated GATA1 expression in K-562. siRNA-mediated knockdown of GATA1 in K-562 cells resulted in reduced TBX1 expression. Western Blot analysis showed reduction GATA1 and TBX1 at the protein level after knockdown (below). (**D**) ChIP-seq data obtained from the ENCODE project showing binding of GATA1 in the upstream region of TBX1. Statistical significance was assessed by Student’s *t*-test (two-tailed) and the calculated *p*-values were indicated by asterisks (*** *p* < 0.001, n.s. not significant).

To identify potential TFs contributing to TBX1 expression in CML tumor cells, we screened TF binding sites given by the UCSC genome browser. This approach revealed several binding sites for multiple TFs located at the TBX1 locus, which may be involved in its transcriptional regulation (Figure 3A). Here, we focused on STAT5 and GATA1, which carry potential binding sites in the TBX1 upstream region and, reportedly, play pathogenic roles in CML [37,38]. RNA-seq data and RQ-PCR analysis demonstrated elevated expression levels of STAT5A and GATA1 in CML cell line K-562 (Figure 3B,C). siRNA-mediated knockdown experiments in K-562 showed that GATA1 activated TBX1 expression while STAT5A had no impact on its regulation (Figure 3B,C). Moreover, analysis of ChIP-seq data from the ENCODE project indicated that GATA1 binds at the corresponding site upstream of TBX1 (Figure 3D). Thus, GATA1 represents an activating TF of TBX1 in CML that directly binds at its regulatory region.

**Figure 4:**
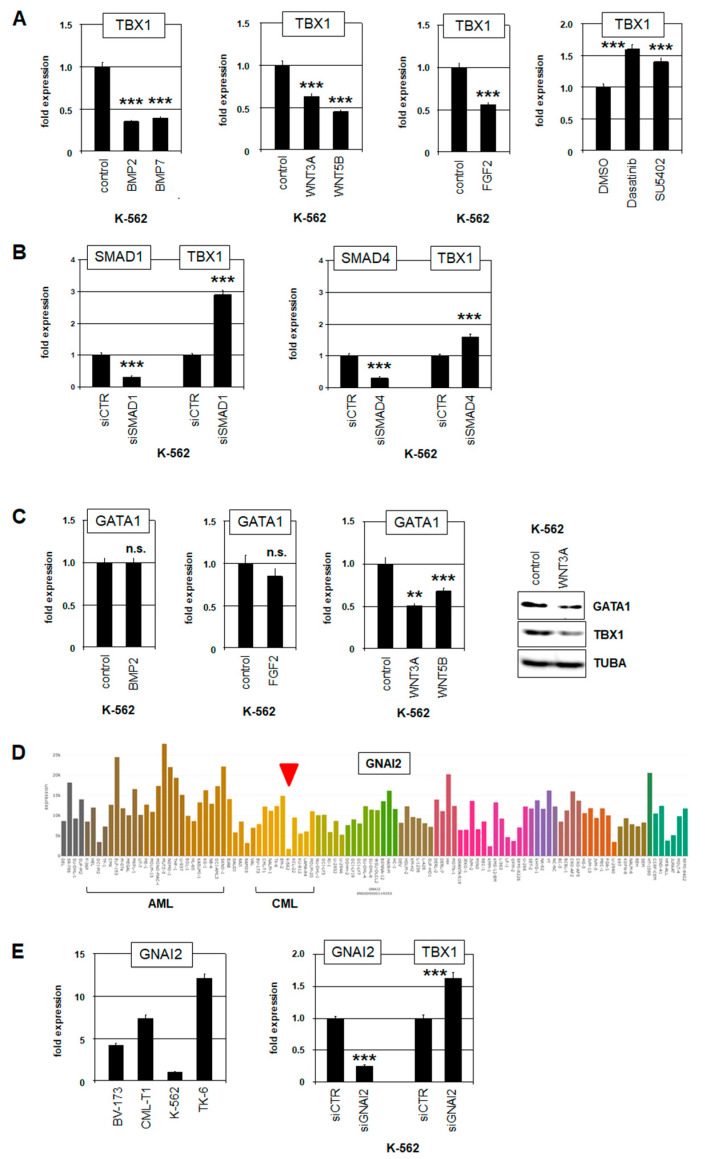
Regulation of TBX1 in CML cell line K-562 by signaling pathways and GNAI2. (**A**) Treatment of K-562 cells with BMP2, BMP7, WNT3A, WNT5B, and FGF2 mediated repression of TBX1 expression, as analyzed by RQ-PCR. Treatment of K-562 cells with ABL1-inhibitor dasatinib or FGF-receptor-inhibitor SU5402 resulted in elevated TBX1 expression. (**B**) siRNA-mediated knockdown of SMAD1 (left) and SMAD4 (right) resulted in elevated TBX1 expression, as analyzed by RQ-PCR. (**C**) RQ-PCR analysis of GATA1 in K-562 cells treated with BMP2, FGF2, WNT3A, and WNT5B. Western Blot analysis demonstrated reduced GATA1 and TBX1 protein levels after treatment with WNT3A (right). (**D**) RNA-seq expression data for GNAI2 in 100 leukemia/lymphoma cell lines showing reduced levels in K-562 (red arrowhead). AML and CML cell lines are indicated. (**E**) RQ-PCR analysis of GNAI2 in CML cell lines confirmed low expression levels in K-562 (left). siRNA-mediated knockdown of GNAI2 in K-562 cells resulted in elevated TBX1 expression (right). Statistical significance was assessed by Student’s *t*-test (two-tailed) and the calculated *p*-values were indicated by asterisks (** *p* < 0.01, *** *p* < 0.001, n.s. not significant).

To identify additional regulators of TBX1 in CML, we performed comparative gene expression profiling analyses. We used dataset GSE57083, comparing TBX1-positive K-562 versus TBX1-negative EM-2, KU-812, and MEG-01 CML cell lines, and dataset GSE44589, comparing 10 TBX1-high versus 10 TBX1-low CML patients. Application of the associated bioinformatic online tool GEOR revealed 250 genes for each comparison, which significantly differ in their expression. Reduced expression levels of signaling pathway genes in TBX1-positive cell lines and patient samples indicated a suppressive role for BMP, WNT, and FGF signaling in TBX1 regulation (Figure S9). Therefore, we treated K-562 cells with BMP2, BMP7, WNT3A, WNT5B, and FGF2 for 20 h. Subsequent RQ-PCR analysis confirmed that all these ligands and pathways inhibition of TBX1 transcription (Figure 4A). Detailed analysis of the BMP-signaling factors SMAD1 and SMAD4 using an siRNA-mediated knockdown approach showed that both factors are involved in TBX1 repression (Figure 4B). Furthermore, treatment of K-562 cells with the FGF-receptor inhibitor SU5402 resulted in elevated TBX1 expression (Figure 4A), corresponding to the results obtained after FGF2 stimulation. Thus, BMP, WNT, and FGF2 signaling mediated repression of TBX1 while their observed downregulation contributed to TBX1 activation in CML.

Next, we analyzed the impact of CML hallmark oncogene BCR::ABL1 on TBX1. Surprisingly, treatment of K-562 cells with ABL1-inhibitor dasatinib resulted in elevated TBX1 expression (Figure 4A). Thus, BCR::ABL1 mediates inhibition of TBX1 expression. This observation corresponded with published data showing that BCR::ABL1 inhibits GATA1 [38], which, in turn, as TBX1 activator (Figure 3C).

To examine whether GATA1 is also involved in the repressive activity of signaling pathways analyzed, we quantified GATA1 expression after treatment of K-562 cells with BMP2, FGF2, WNT3A, and WNT5B. The data indicated that WNT3A and WNT5B mediated inhibition of GATA1 transcription while BMP2 and FGF2 showed no significant effect on the expression of this factor (Figure 4C).

Finally, GNAI2 reportedly regulates TBX1 in developing muscle cells [39]. RNA-seq data and RQ-PCR analysis indicated decreased GNAI2 expression in K-562 (Figure 4D,E). siRNA-mediated downregulation of GNAI2 in K-562 cells resulted in elevated TBX1 expression (Figure 4E), demonstrating that GNAI2 performs TBX1 inhibition and its downregulation in K-562, thus, stimulates TBX1 expression. A comparison of selected gene activities in 93 leukemia cell lines using data from the Human Protein Atlas showed elevated expression of TBX1 and GATA1 and low levels of GNAI2 in K-562 (Figure S10), confirming our findings.

Taken together, analysis of model cell line K-562 revealed several regulators of TBX1 in CML. GATA1 was identified as a key activator, which is highly expressed in K-562 and negatively regulated by WNT signaling and BCR::ABL1. TBX1 repressor GNAI2 was downregulated in K-562, thus contributing indirectly to elevated expression levels.

### 2.4. Target Genes of TBX1 in CML

**Figure 5:**
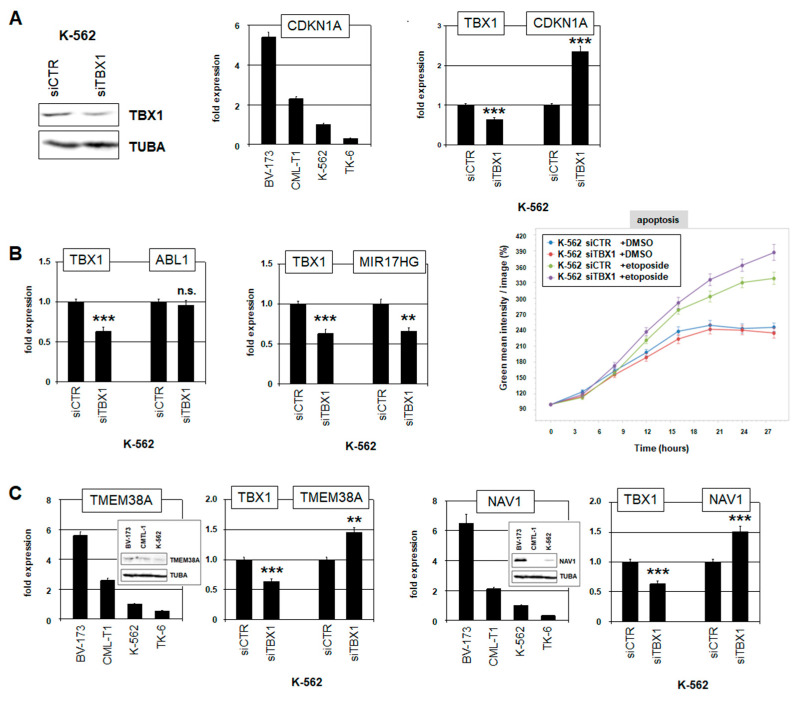
Target genes and oncogenic function of TBX1 in CML. (**A**) Western Blot analysis of TBX1 in K-562 cells treated for siRNA-mediated knockdown (left). RQ-PCR analysis of CDKN1A in CML cell lines, showing reduced expression levels in TBX1-positive cell lines K-562 and TK-6 (middle). RQ-PCR analysis of CDKN1A after siRNA-mediated knockdown of TBX1 in K-562 cells (right). (**B**) RQ-PCR analysis of ABL1 (left) and MIR17HG (middle) after siRNA-mediated knockdown of TBX1 in K-562 cells. Live-cell imaging analysis of K-562 cells treated for siRNA-mediated knockdown of TBX1 with etoposide, showing apoptosis levels (right). (**C**) RQ-PCR and Western Blot analyses (insert) of TMEM38A and NAV1 in CML cell lines. RQ-PCR analyses of TMEM38A and NAV1 in K-562 cells treated for siRNA-mediated knockdown of TBX1. Statistical significance was assessed by Student’s *t*-test (two-tailed) and the calculated *p*-values were indicated by asterisks (** *p* < 0.01, *** *p* < 0.001, n.s. not significant).

Next, we investigated the role and oncogenic function of TBX1 in CML. TBX1 plays a fundamental role in developing muscle cells, regulating their proliferation via CDKN1A [40]. Downregulation of cell cycle inhibitor CDKN1A is a hallmark of many cancer types [41]. Thus, we performed siRNA-mediated knockdown of TBX1 in K-562 cells to study its regulatory impact in CML (Figure 5A). CDKN1A showed low expression levels in TBX1-positive K-562 and TK-6 cells (Figure 5A), supporting its tumor suppressor status in this disease. Furthermore, TBX1 knockdown resulted in elevated CDKN1A levels (Figure 5A), demonstrating that TBX1 repressed CDKN1A in CML.

TBX1 knockdown showed no significant effect on ABL1 expression (Figure 5B), discounting mutual regulation. However, TBX1 drove activation of the micro-RNA host gene MIR17HG (Figure 5B), which has been reported as key anti-apoptotic target of BCR::ABL1 in CML [42]. In correspondence to this finding, live-cell imaging analysis of K-562 cells treated for TBX1 knockdown showed elevated levels of apoptosis, which raised even further after additional treatment with etoposide (Figure 5B). Thus, TBX1 supported survival of CML cells, possibly mediated via MIR17HG activation.

To identify additional TBX1 target genes in CML, we performed RNA-sequencing of K-562 cells after siRNA-mediated knockdown of TBX1. This exercise revealed 21 genes were significantly activated and 12 genes repressed by TBX1 (Table S1). For detailed analysis, we focused on TMEM38A and NAV1 being targeted for repression. Public expression data from the Human Protein Atlas showed elevated activities of TMEM38A and NAV1 in mature myeloid cells from healthy donors, notably basophils and monocytes, and, additionally, of NAV1 in myeloid/conventional DCs (Figure S11), indicating disturbed myeloid differentiation when these genes are suppressed by TBX1. These findings were supported by RQ-PCR and Western Blot analyses, which demonstrated low expression levels of TMEM38A and NAV1 in K-562 (Figure 5C). Furthermore, RQ-PCR analysis of K-562 cells revealed elevated expression levels of both genes after TBX1 knockdown (Figure 5C). We detected two potential TBX1 binding sites in Intron 1 of TMEM3A and 16 binding sites in different introns of NAV1, supporting that TBX1 regulates these genes directly. Thus, we identified myeloid-associated TMEM38A and NAV1 as novel repressed target genes of TBX1 in CML.

**Figure 6:**
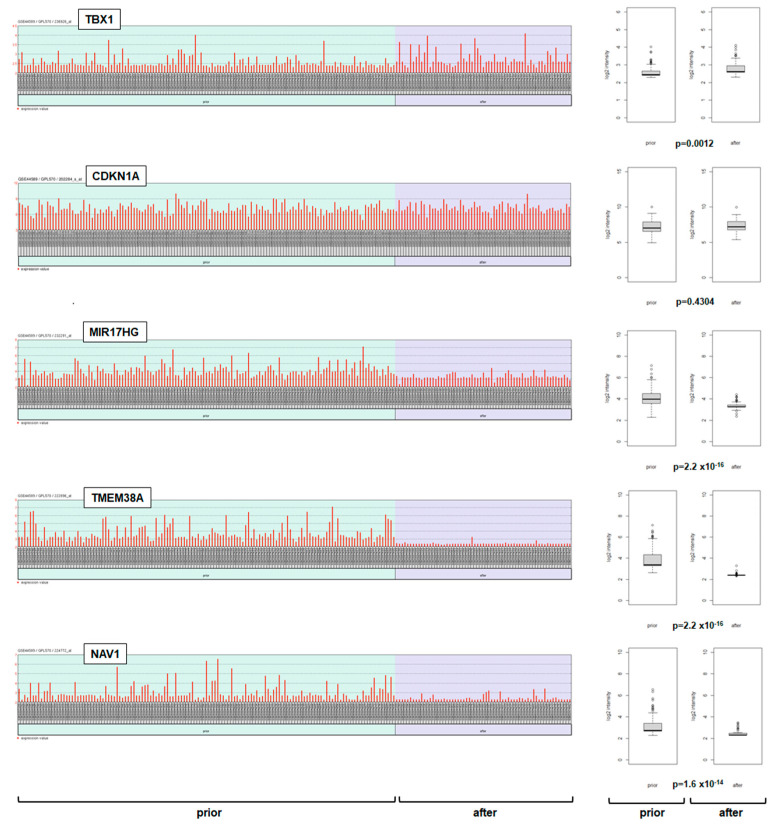
Impact of imatinib on TBX1 and its target genes in CML patients. Barplots show the activities of TBX1 and its target genes, CDKN1A, MIR17HG, TMEM38A, and NAV1, obtained from gene expression profiling dataset GSE44589, which covers 198 CML patients prior to (light green background) and after (light blue background) treatment with imatinib (left). Additionally, these patient groups were statistically compared, as shown by boxplots on the right. The resulting *p*-values are indicated.

Finally, we analyzed TBX1 and its target genes, identified here using gene expression profiling dataset GSE44589, which covers 198 CML patients prior to and after treatment with ABL1-inhibitor imatinib. The data showed that TBX1 expression was significantly higher in patients treated with imatinib (Figure 6), confirming our results K-562 cells treated with ABL1-inhibitor dasatinib (Figure 4A). However, CDKN1A expression levels did not correspond to those of TBX1 (Figure 6), which may indicate that factors independent of ABL1 and TBX1 regulate CDKN1A in CML as well. In contrast, MIR17HG, and more so TMEM38A and NAV1, showed reduced expression levels after imatinib treatment (Figure 6). MIR17HG is reportedly activated by BCR::ABL1 [42]. Thus, its downregulation after imatinib treatment may result from both ABL1 inhibition and TBX1 derepression. imatinib-mediated reduction TMEM38A and NAV1 correlated with elevated TBX1 (Figure 6), thus reflecting the repressive impact of TBX1 on these genes.

Taken together, TMEM38A and NAV1 apparently represent key target genes deregulated by TBX1 in CML. Treatment with imatinib inhibits BCR::ABL1 but enhances TBX1 expression and consequently downregulates the myeloid genes TMEM38A and NAV1.

## 3. Discussion

**Figure 7:**
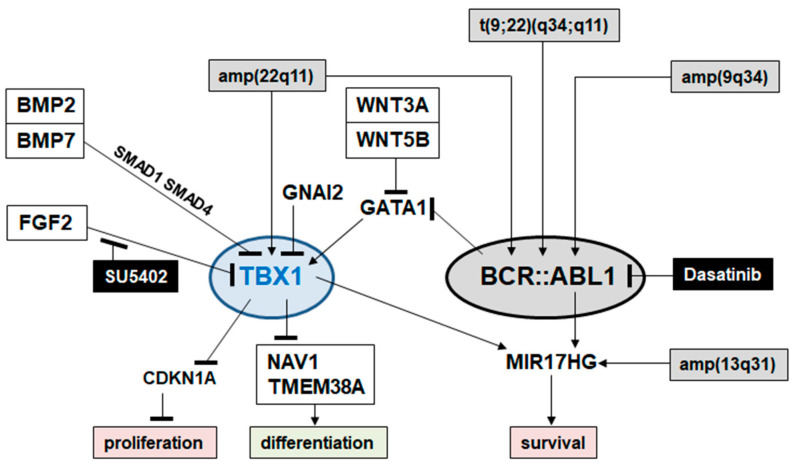
Gene regulatory network of TBX1 in CML, showing genomic aberrations, activating factors, signaling pathways, pharmacological inhibitors, and downstream targets and functions.

In this study, we have established the myeloid TBX-code, representing a collective gene signature that describes physiological activities of 10 T-box TF encoding genes in early hematopoiesis and in myelopoiesis. We used this code to identify deregulated T-box genes in myeloid malignancies, including AML and CML. Detailed investigations focused on TBX1, which showed normal activity in hematopoietic progenitors and aberrant activation in 10% of CML patients. TBX1-expressing CML cell line K-562 served as a model to investigate upstream and downstream factors of TBX1. These results are summarized in Figure 7, depicting the gene regulatory network around TBX1. This pathogenic network may help to establish novel markers and therapeutic targets for clinical diagnostics and treatment of CML subsets, respectively.

We introduced the concept of TF-codes for the hematopoietic compartment analyzing the TF encoding group of NKL homeobox genes and extended this approach to TALE homeobox genes, ETS genes, and T-box genes. Furthermore, we have exploited the generated signatures to identify deregulated TFs in particular hematopoietic malignancies, including T-cell acute lymphoid leukemia, anaplastic large cell lymphoma (ALCL), and Hodgkin lymphoma [10,20,43]. According to those studies, this concept proved to illuminate transcriptional regulation of normal hematopoiesis and the role of deregulated TFs in leukemia/lymphoma.

Here, we examined T-box TFs in myelopoiesis, focusing on TBX1 in CML. T-box factors encode developmental TFs, which reportedly promote developmental diseases and cancer if mutated or deregulated [11]. TBX1 is physiologically expressed in mesodermal progenitors, in the pharyngeal region during embryogenesis, in developing heart and muscles, and in beige adipocytes [11,44,45]. Genomic deletions at chromosomal region 22q11, which includes the TBX1 locus, have been reported in patients with DiGeorge syndrome, supporting its role in the development of the pharyngeal region and the heart and highlighting copy number alterations in deregulation [46]. Accordingly, we detected genomic amplification of TBX1 in CML cell lines K-562 and TK-6. TBX1 was found to be aberrantly activated in 10% of CML patients. CML tumor cells carry the hallmark fusion gene BCR::ABL1, which is generated by chromosomal rearrangement t(9;22)(q34;q11) [32,33]. The design of ABL1 inhibitors imatinib and dasatinib represented milestones for the therapy of CML. However, the appearance of resistance to these drugs limits their efficacy and demands alternative therapeutic targets [35]. Our study may support this task.

The TBX1-related gene org-1 plays conserved roles in mesodermal muscle and heart development in the fruit fly *Drosophila melanogaster* [47,48]. Org-1 has been described as a target of an ALK-related gene [49], forming a regulatory connection to tyrosine kinases. However, ALK-positive ALCL cell lines were TBX1-negative and BCR::ABL1 performed inhibition of TBX1 expression in K-562. Thus, tyrosine-kinase-mediated activation of TBX1 is apparently absent in malignant hematopoietic cells. However, TBX1 and BCR are genomic neighbors at chromosomal position 22q11. Consequently, a genomic amplification at 22q11 (and additionally at 9q34) in K-562 cells targets both TBX1 and fusion gene BCR::ABL1 [21], highlighting their pathogenic function in CML.

TF binding site analysis and functional tests revealed that GATA1 is an activator of TBX1 expression in CML cells. However, GATA1 is reportedly inhibited by BCR::ABL1 and, in turn, downregulates BCR::ABL1 via miR138 [38]. Accordingly, GATA1 is weakly expressed in CML but elevated in essential thrombocythemia and polycythemia vera—all myeloproliferative neoplasias [50]. In zebrafish, GATA4/5/6 activate TBX1 in heart development while aberrant activity of GATA6 underlies heart defects [51,52], thus supporting a conserved role for GATA factors in TBX1 activation. Furthermore, we detected that the WNT-signaling pathway suppressed TBX1 via inhibition of GATA1 while BMP and FGF signaling performed TBX1 inhibition without regulation of GATA1. TBX1 inhibition by BMP signaling has also been described in beige adipocytes [45], highlighting this kind of regulation for TBX1 expression. However, the BMP/SMAD1/4 pathway and WNT signaling regulate GATA1 in hematopoiesis [53], indicating context-dependent differences in their downstream activities. Finally, we showed that GNAI2 suppressed TBX1 expression in K-562. In contrast, GNAI2 activates TBX1 in developing muscle cells [39]. GNAI2 encodes a G-protein implicated in an adenylate-cyclase-containing signaling pathway which, therefore, performs inhibitory activity in CML cells.

Our analyses of TBX1 downstream activities revealed four target genes, including activated MIR17HG, and repressed CDKN1A, TMEM38A, and NAV1. MIR17HG is a reported TBX1 target during heart development [54] and BCR::ABL1 activates MIR17HG in CML [42]. MIR17HG is a potent oncogene in hematopoietic malignancies [55], thus highlighting the oncogenic role of TBX1 in CML. Furthermore, MIR17HG inhibits the anti-apoptotic factor BCL2L11/BIM, which is activated by etoposide, thus enhancing the apoptotic effect of TBX1 knockdown in K-562 [55,56]. CDKN1A encodes the cell cycle inhibitor p21 and serves as a tumor suppressor [41]. CDKN1A is a reported target gene in a mouse TBX1 deletion model of the DiGeorge syndrome [40], highlighting CDKN1A as a general target of TBX1. Of note, TBX1 may regulate its target genes directly or indirectly via interaction with chromatin factors [44]. concordant consensus sequence for DNA interaction and histone methyltransferases as chromatin binding partners have been reported [57,58].

Furthermore, we identified two hitherto unreported genes suppressed by TBX1, namely, TMEM38A and NAV1. Both genes are physiologically upregulated in mature myeloid cells, indicating a role in myeloid differentiation (Figure S11). TMEM38A is also expressed in muscles, suggesting its regulation by TBX1 in this tissue as well (Figure S11). However, the regulatory impact in CML is repressive, suggesting different cofactors may be involved. TMEM38A encodes a Ca^2+^ channel, enhancing cytosolic Ca^2+^ levels [59]. Consistent with this proposal, CML cells have been shown to contain reduced Ca^2+^ levels [60]. Thus, reduced expression of TMEM38A mediates decreased concentrations of Ca^2+^ in the tumor cells. Furthermore, TMEM38A regulates the development of muscle cells via organization of the nuclear peripheral region [61], which may also play a role in myeloid differentiation. Finally, NAV1 encodes a cytoskeletal-associated protein is highly expressed in the heart [62], suggesting that TBX1 may regulate NAV1 in this tissue (Figure S11). Our data revealed additional elevated NAV1 expression levels in DCs (Figure S11). Functionally, NAV1 is involved in micropinocytosis [62]. This activity plays a role in antigen presentation, which, in turn, is reportedly downregulated in DCs from CML [63]. Thus, TBX1 may disturb the function of DCs in CML patients via NAV1 repression.

Taken together, identification of aberrantly activated TBX1 and its deregulated target genes contributes to the understanding of the pathogenesis of CML and may assist in developing novel therapies. However, the roles of TMEM38A and NAV1 in CML require additional investigation before qualifying as actionable targets. These studies are currently ongoing in our institute.

## 4. Materials and Methods

### 4.1. Bioinformatic Analyses of Gene Expression Data

Screening for T-box gene activities in entities of normal myelopoiesis and mature immune cells was performed using public gene expression data, including RNA-sequencing data from the Human Protein Atlas (HPA, www.proteinatlas.org) and gene expression profiling data obtained from Gene Expression Omnibus (GEO, www.ncbi.nlm.nih.gov, accessed on 1 October 2023), namely, GSE42519 (for myelopoiesis), GSE22552 (erythropoiesis), GSE109348 (monocytes, mast cells, granulocytes, monocyte-derived dendritic cells), and GSE40831 (megakaryocytes). Patients were analyzed using public gene expression profiling data from AML patients (GSE15434 and GSE14468) and CML patients (GSE44589). exploitation of cell lines was performed using RNA-sequencing data from 100 leukemia/lymphoma cell lines (termed LL-100), available at ArrayExpress (www.ebi.ac.uk/arrayexpress, accessed on 1 October 2023) via E-MTAB-7721, which was vizualised using R/Bioconductor tools DESeq2 and shinyngs (https://github.com/pinin4fjords/shinyngs, accessed on 1 October 2023) and gene expression profiling dataset GSE57083. TF binding site analysis was performed using the UCSC genome browser (www.genome.cse.ucsc.edu, accessed on 1 October 2023). Direct TF binding was analyzed using ChIP-seq data from the ENCODE project (www.genome.gov). RNA-sequencing data from siRNA-treated K-562 cells (performed in triplicate) were generated at Eurofins MWG (Ebersberg, Germany). Sample libraries for control and treated K-562 cells were prepared with the strand-specific cDNA library and sequenced 2 × 150 bp by Eurofins Genomics on the Illumina NovaSeq 6000 platform (INVIEW Transcriptome, Ebersberg, Germany) by aiming for a minimum of 30 M reads per sample with an insert size of >150 bp. Trimming and quality control of the sequencing reads were performed via fastp [64] and quantification of the reads via salmon [65] on the reference human gencode GRCh38, version 37. Finally, data were analyzed with DESeq2 [66], R/Bioconductor. The data are available from BioStudies (www.ebi.ac.uk/biostudies, accessed on 1 October 2023) via S-BSST1176. For analysis of TBX1 binding sites, we used the CIS-BP database (www.cisbp.ccbr.utoronto.ca/index.php, accessed on 1 October 2023) and the UCSC genome browser.

### 4.2. Cell Lines and Treatments

Cell lines are held by the DSMZ (Braunschweig, Germany) and cultivated as described on the website (www.dsmz.de, accessed on 1 October 2023). All cell lines had been authenticated and tested negative for mycoplasma infection. Modification of gene expression levels was performed using gene-specific siRNA oligonucleotides, with reference to AllStars negative Control siRNA (siCTR) obtained from Qiagen (Hilden, Germany). siRNA (80 pmol) was transfected into 1 × 10^6^ cells by electroporation using the EPI-2500 impulse generator (Fischer, Heidelberg, Germany) at 350 V for 10 ms. Cell lines were also treated with 20 ng/mL BMP2, BMP7, WNT3A, WNT5B, and FGF2 (R&D Systems, Wiesbaden, Germany) and with 100 µM dasatinib, 10 µM SU5402, and 5 µM 3-Deazaneplanocin A (DZNep), all obtained from Sigma (Taufkirchen, Germany). Electroporated and treated cells were performed twice and harvested after 20 h cultivation.

Apoptosis was analyzed using the IncuCyte S3 Live-Cell Analysis System (Essen Bioscience, Hertfordshire, UK) combined with the IncuCyte Caspase-3/7 Green Apoptosis Assay diluted at 1:2000 (Essen Bioscience, Essen, Germany). Cells were additionally treated with 100 µM etoposide (Sigma, Taufkirchen, Germany) dissolved in dimethyl sulfoxide (DMSO). Finally, the concentration of DMSO was 0.1%. Live-cell imaging experiments were performed twice with fourfold parallel tests.

### 4.3. Polymerase Chain Reaction (PCR) Analysis

Total RNA was extracted from cultivated cell lines using TRIzol reagent (Invitrogen, Darmstadt, Germany). Primary human total RNA derived from CD34-positive hematopoietic stem cells (HSCs) and peripheral mononuclear blood cells (PBCs) was purchased from Miltenyi Biotec (Bergisch Gladbach, Germany)., cDNA was synthesized using 1 µg RNA, random priming, and Superscript II (Invitrogen). Real-time quantitative (RQ)-PCR analysis was performed using the 7500 Real-time System and commercial buffer and primer sets (Applied Biosystems/Life Technologies, Darmstadt, Germany). For normalization of expression levels, we quantified the transcripts of TATA box binding protein (TBP), applying the delta-delta-Ct method. For quantification of TBX1 genomic copy numbers, we used the following oligonucleotides: TBX1-1 5′-GGACATGTCCTGAAGGACAAGG-3′ and TBX1-2 5′-GTATTGAAGGGTTGGCACTCTGC-3′. The locus of MEF2C was used as control: MEF2C-1 5′-GCAGGAATTTGGGAACTGAG-3′ and MEF2C-2 5′-CCCATAGTCCCCGTTTTTCT-3′. Oligonucleotides were obtained from Eurofins MWG (Ebersberg, Germany). Genomic DNA was prepared as described above. Quantitative analyses were performed as biological replicates and measured in triplicate. Standard deviations are presented in the figures as error bars. Statistical significance was assessed by Student’s *t*-test (two-tailed) and the calculated *p*-values were indicated by asterisks (* *p* < 0.05, ** *p* < 0.01, *** *p* < 0.001, n.s. not significant).

### 4.4. Protein Analysis

Western Blots were performed by the semi-dry method. Protein lysates from cell lines were prepared using SIGMAFast protease inhibitor cocktail (Sigma, Taufkirchen, Germany). Proteins were transferred onto nitrocellulose membranes (Bio-Rad, Munich, Germany) and blocked with 5% dry milk powder dissolved in phosphate-buffered saline (PBS) buffer. The following antibodies were used: alpha-tubulin (Sigma, #T6199), GATA1 (Cell Signaling, Danvers, MA, USA, #D52H6), SMAD1 (Santa Cruz Biotechnology, Heidelberg, Germany, #sc-7965), TBX1 (Origene, Rockville, MD, USA#TA347536), TMEM38A (Santa Cruz Biotechnology, Heidelberg, Germany, #sc-390054), NAV1 (MyBioSource, San Diego, CA, USA, #MBS9419483). For loading control, blots were reversibly stained with Ponceau (Sigma); the detection of alpha-tubulin (TUBA) was performed thereafter. Secondary antibodies were linked to peroxidase for detection by Western Lightning ECL (Perkin Elmer, Waltham, MA, USA). Documentation was performed using the digital system ChemoStar Imager (INTAS, Göttingen, Germany).

### 4.5. Karyotyping and Genomic Profiling Analysis

Cytogenetic analyses (karyotyping, FISH, and SKY) were performed as described previously [67]. Generated karyotypes of analyzed cell lines are provided at www.dsmz.de. For genomic profiling, genomic DNA of AML cell lines was prepared by the Qiagen Gentra Puregene Kit (Qiagen). labeling, hybridization, and scanning of Cytoscan HD single nucleotide polymorphism (SNP) arrays were performed by the Genome Analytics Facility located at the Helmholtz Centre for Infection Research (Braunschweig, Germany), according to the manufacturer’s protocols (Affymetrix, High Wycombe, UK). Data were interpreted using the Chromosome Analysis Suite software version 3.1.0.15 (Affymetrix, High Wycombe, UK) and copy number alterations determined accordingly.

## 5. Conclusions

We report establishment of the myeloid TBX-code, which represents a signature of 10 T-box genes in myelopoiesis and serves to identify aberrantly expressed T-box genes in myeloid malignancies. Accordingly, TBX1 is a novel oncogene in CML subsets, creating a gene network that spotlights novel biomarkers and potential therapeutic targets in this intractable entity.

## Data Availability

The information on the datasets generated or analyzed during this study are included in this published article and its Supplementary Information Files. The accession codes of all publicly available data are given in the Material and Methods Section.

## Supplementary Materials

The following supporting information can be downloaded at: https://www.mdpi.com/article/10.3390/ijms25010032/s1.
