# Supplementary material for: Establishment of the Myeloid TBX-Code Reveals Aberrant Expression of T-Box Gene TBX1 in Chronic Myeloid Leukemia
Source: Int J Mol Sci. 2023 Dec 19;25(1):32. doi: 10.3390/ijms25010032 (PMC10778679; doi:10.3390/ijms25010032)
Supplement: Supplementary file 1 [file ijms-25-00032-s001.zip › ijms-2755925-supplementary.pdf]

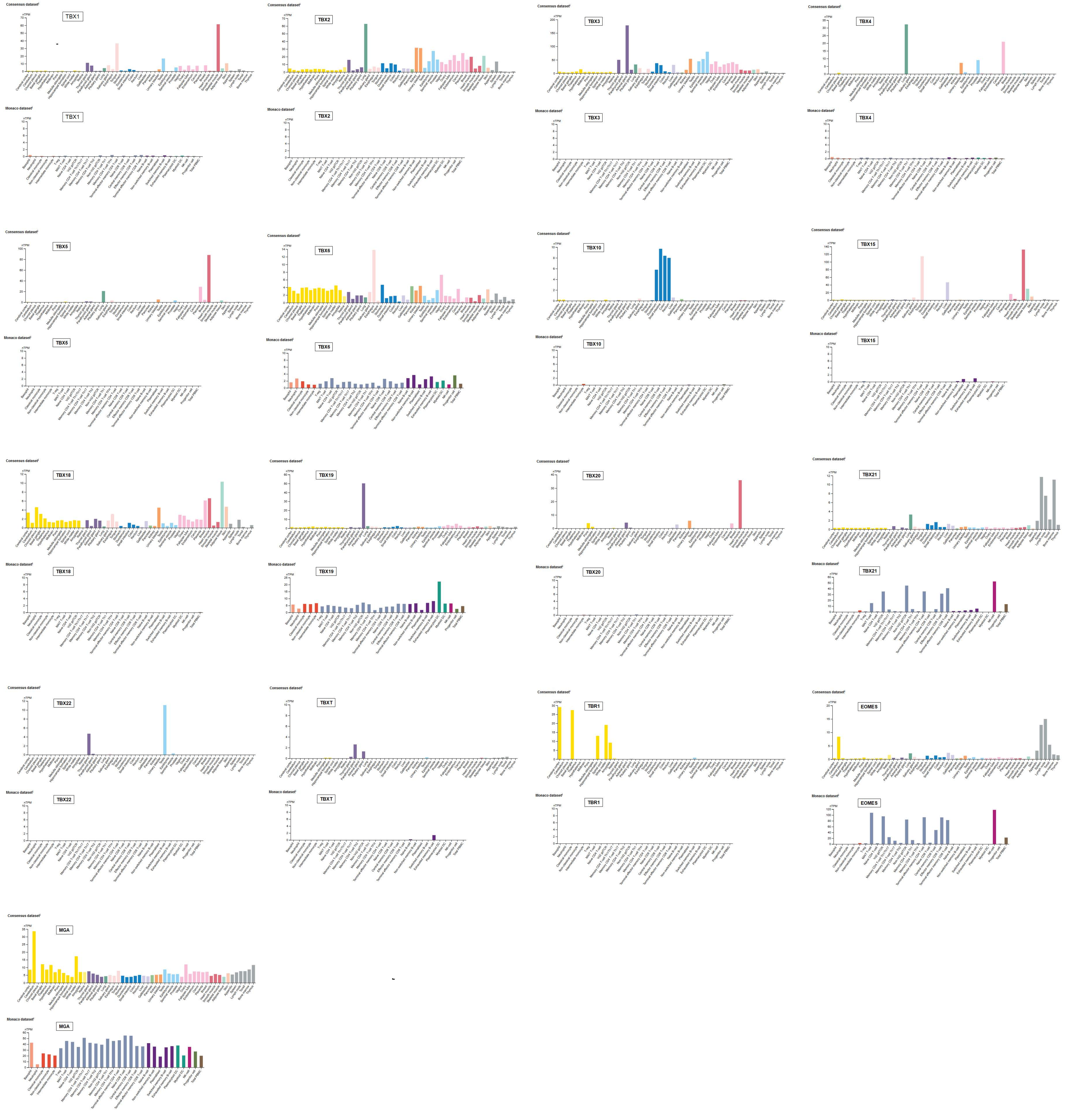

Figure S1: T-box gene activities using RNA-seq data from the Human Protein Atlas for diverse tissue types (Consensus dataset, above) and hematopoietic cell types (Monaco dataset, below).

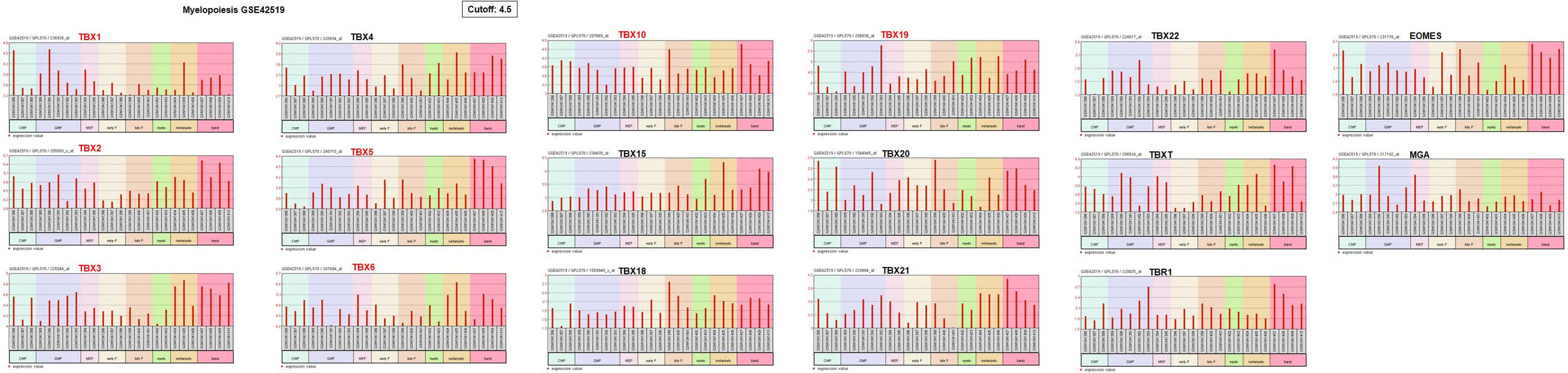

Figure S2: T-box gene activities using dataset GSE42519 for particular stages of myelopoiesis. A cutoff was set at 4.5.

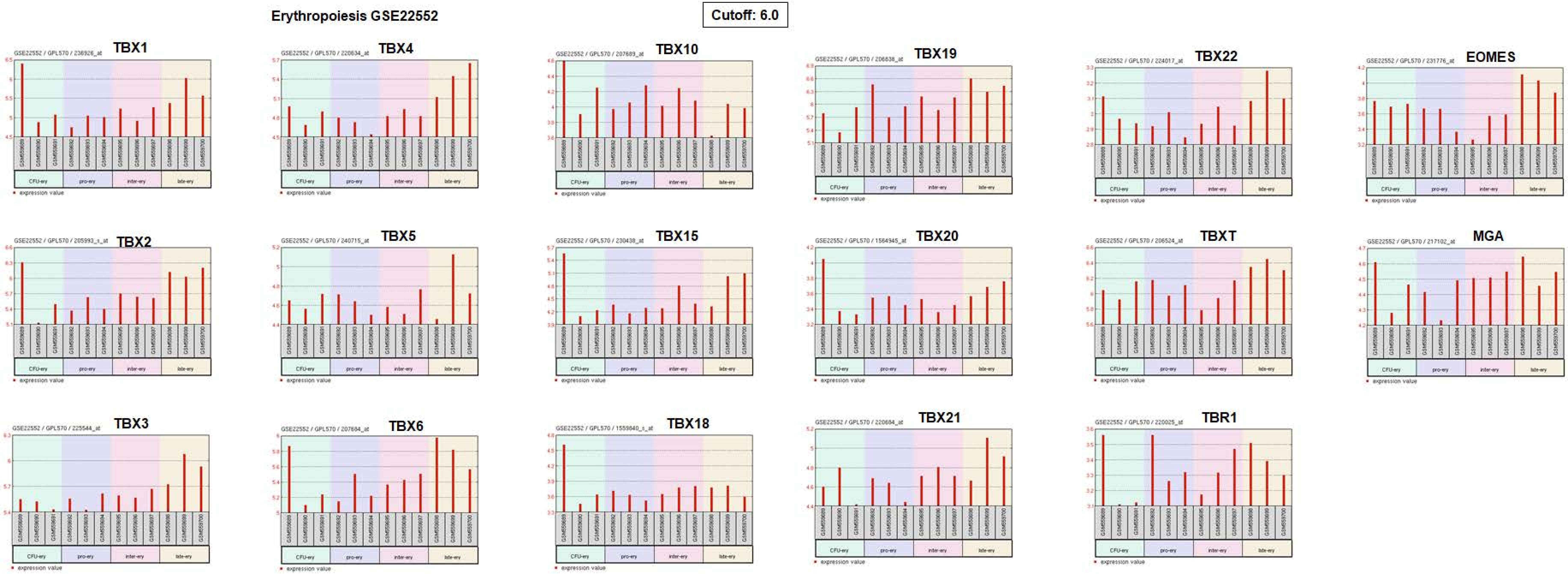

Figure S3: T-box gene activities using dataset GSE22552 for particular stages of erythropoiesis. A cutoff was set at 6.0.

Megakaryocytes GSE40831

Cutoff: 50

Group	Accession	Title	Source name	Cell type
-	GSM1002641	megakaryocytes at day0, biological rep A	umbilical cord blood	CD34-CD41+CD61+CD45- megakaryocytes
-	GSM1002642	megakaryocytes at day0, biological rep B	umbilical cord blood	CD34-CD41+CD61+CD45- megakaryocytes
-	GSM1002643	megakaryocytes at day4, biological rep A	Lin- cell culture derived samples, day4	CD34-CD41+CD61+CD45- megakaryocytes
-	GSM1002644	megakaryocytes at day4, biological rep B	Lin- cell culture derived samples, day4	CD34-CD41+CD61+CD45- megakaryocytes
-	GSM1002645	CFU-M at day0, biological rep A	umbilical cord blood	CD34-CD33+CD13+ colony forming unit-monocytes
-	GSM1002646	CFU-M at day0, biological rep B	umbilical cord blood	CD34-CD33+CD13+ colony forming unit-monocytes
-	GSM1002647	CFU-M at day4, biological rep A	Lin- cell culture derived samples, day4	CD34-CD33+CD13+ colony forming unit-monocytes
-	GSM1002648	CFU-M at day4, biological rep B	Lin- cell culture derived samples, day4	CD34-CD33+CD13+ colony forming unit-monocytes

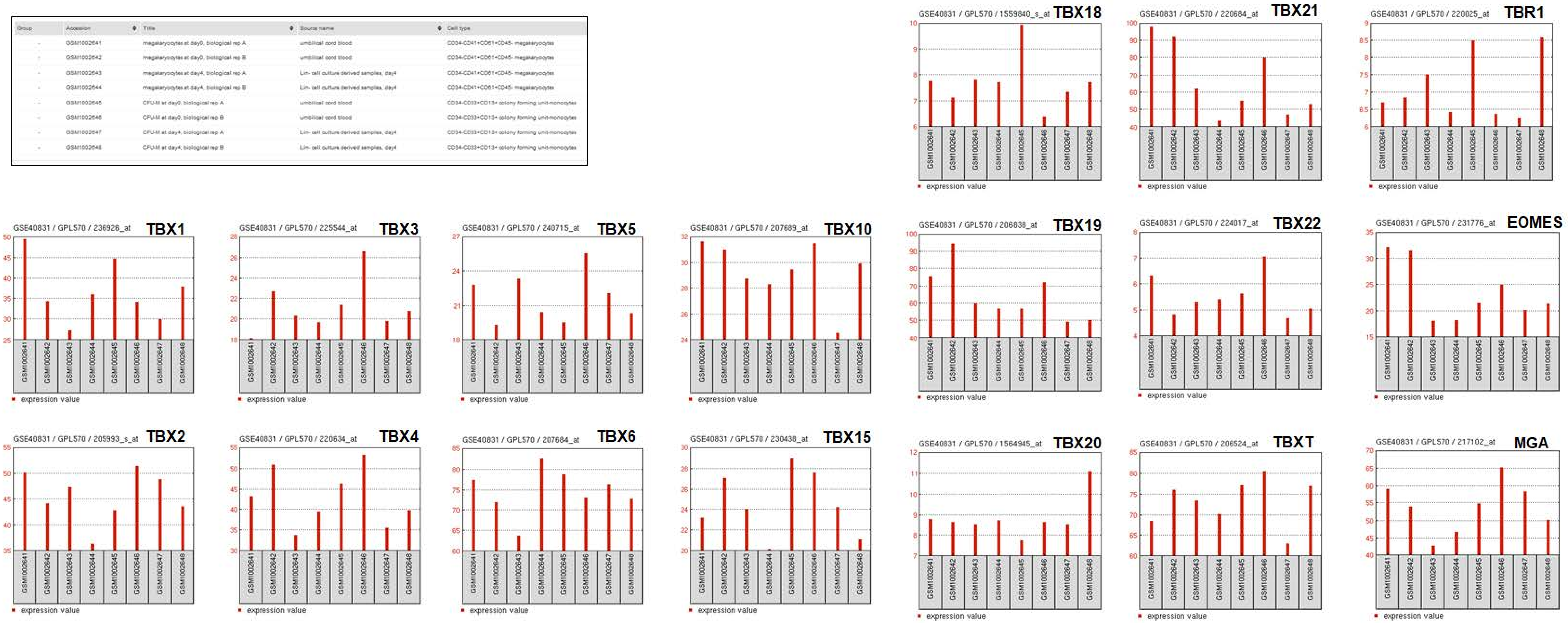

Figure S4: T-box gene activities using dataset GSE40831 for megakaryocytes. A cutoff was set at 50.

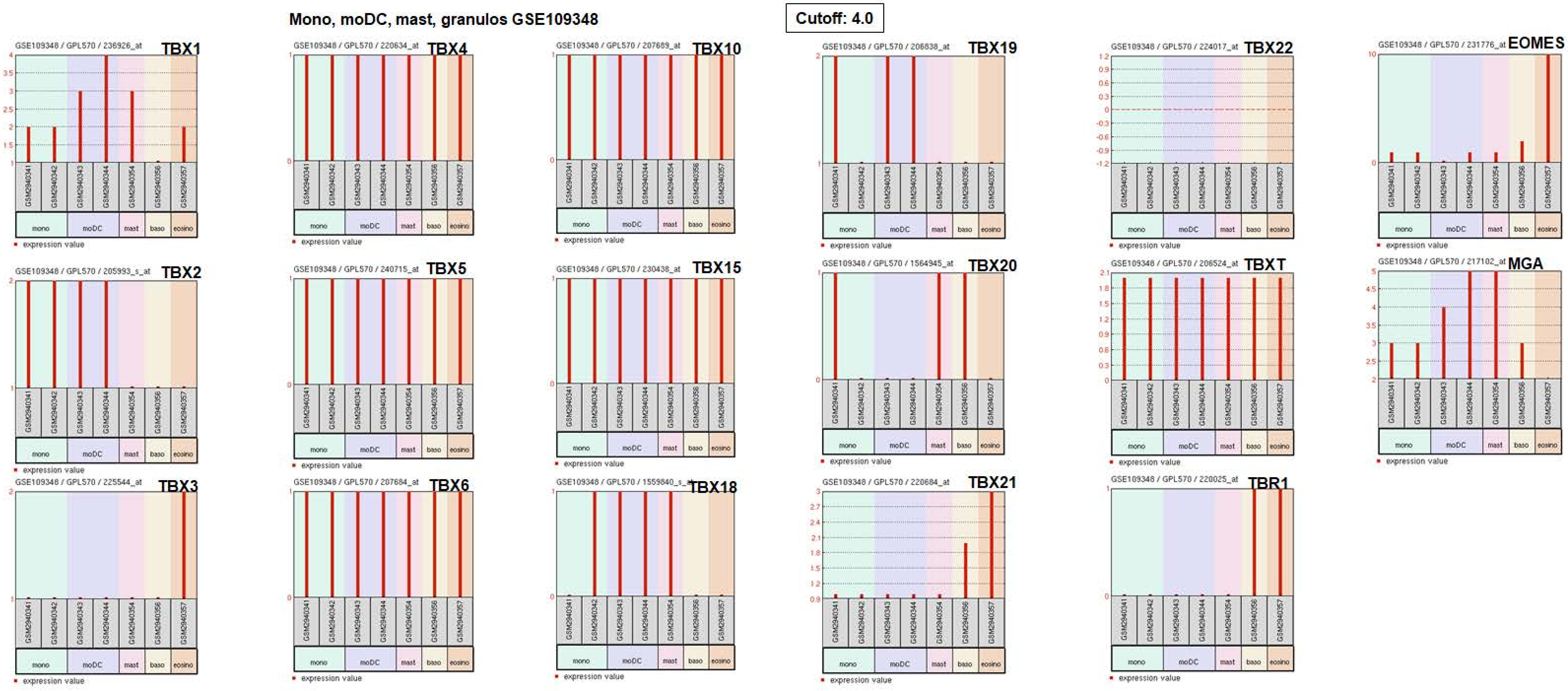

Figure S5: T-box gene activities using dataset GSE109348 for monocytes, monocyte-derived dendritic cells, mast cells and granulocytes. A cutoff was set at 4.0.

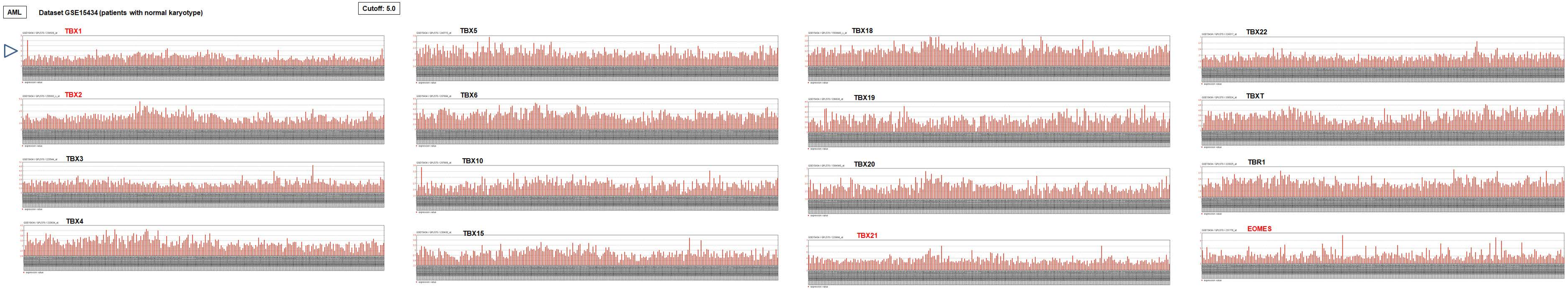

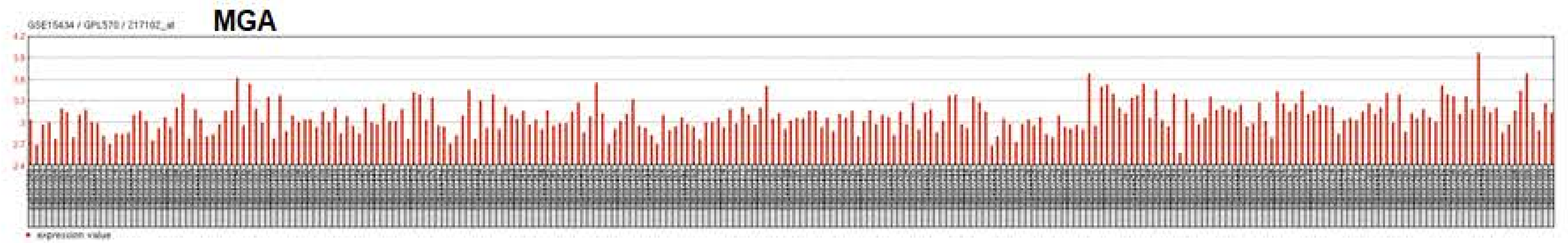

Figure S6: T-box gene activities using dataset GSE15434 for AML patients with normal karyotype. A cutoff was set at 5.0. Aberrantly expressed T-box genes are indicated in red.

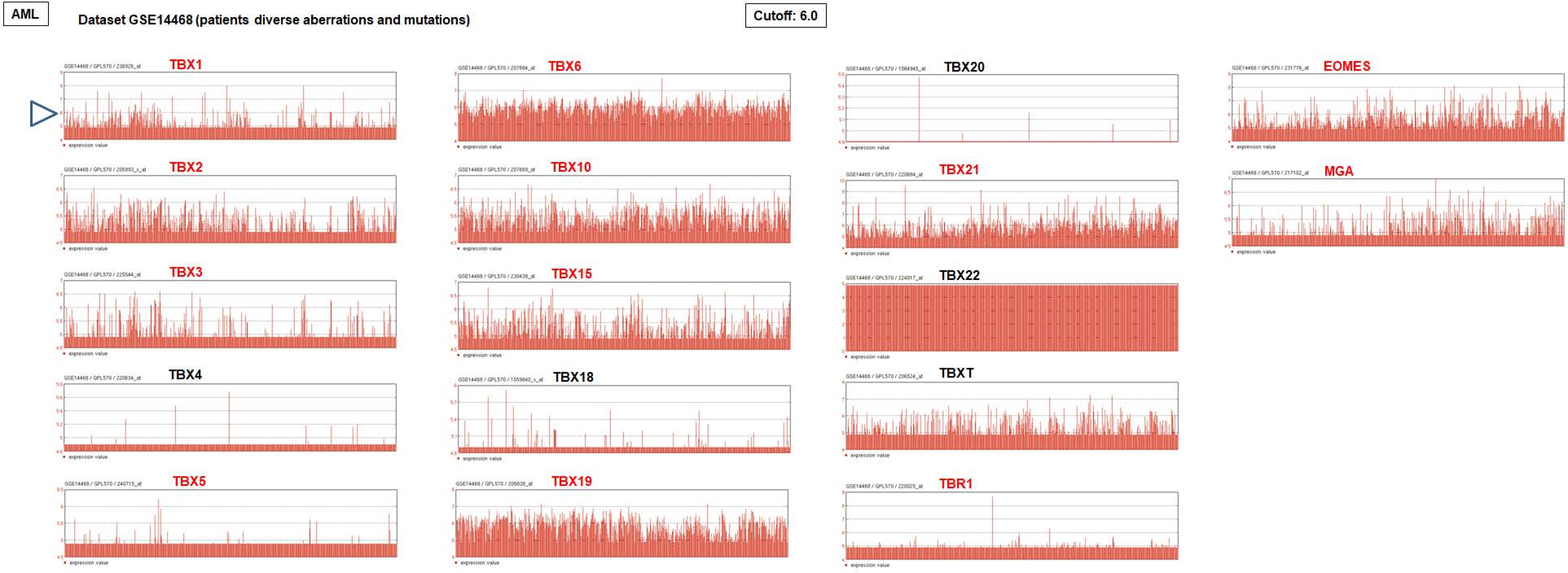

Figure S7: T-box gene activities using dataset GSE14468 for AML patients with diverse aberrations and mutations. A cutoff was set at 6.0. Aberrantly expressed T-box genes are indicated in red.

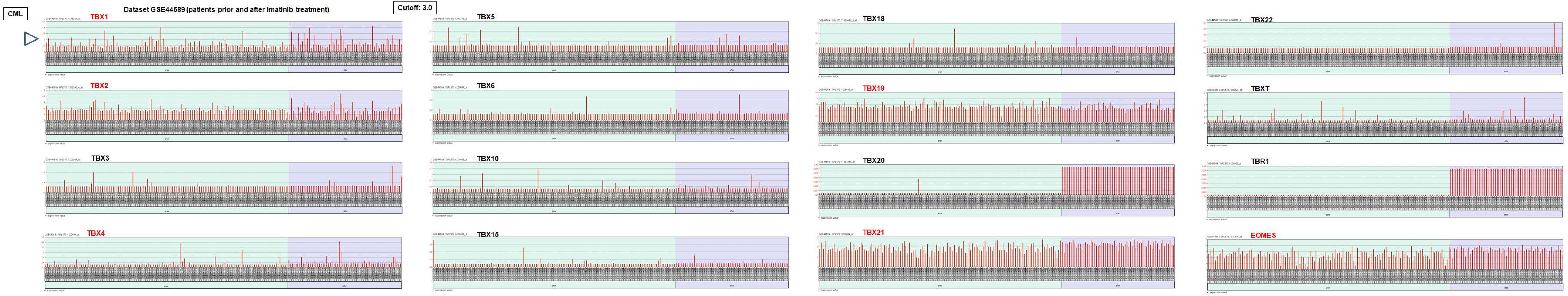

Figure S8: T-box gene activities using dataset GSE44589 for CML patients prior and after imatinib treatment. A cutoff was set at 3.0. Aberrantly expressed T-box genes are indicated in red.

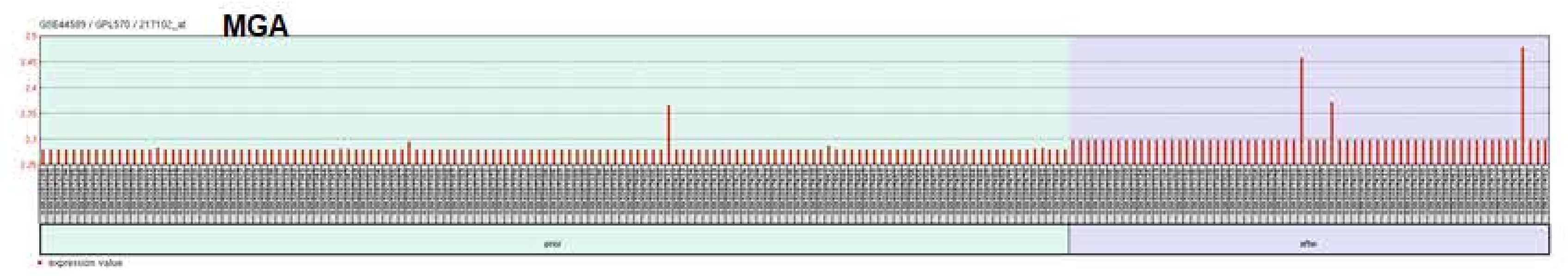

GEOR analysis of **cell line** dataset GSE57083
comparing K-562 versus CML control cell lines (top-250)

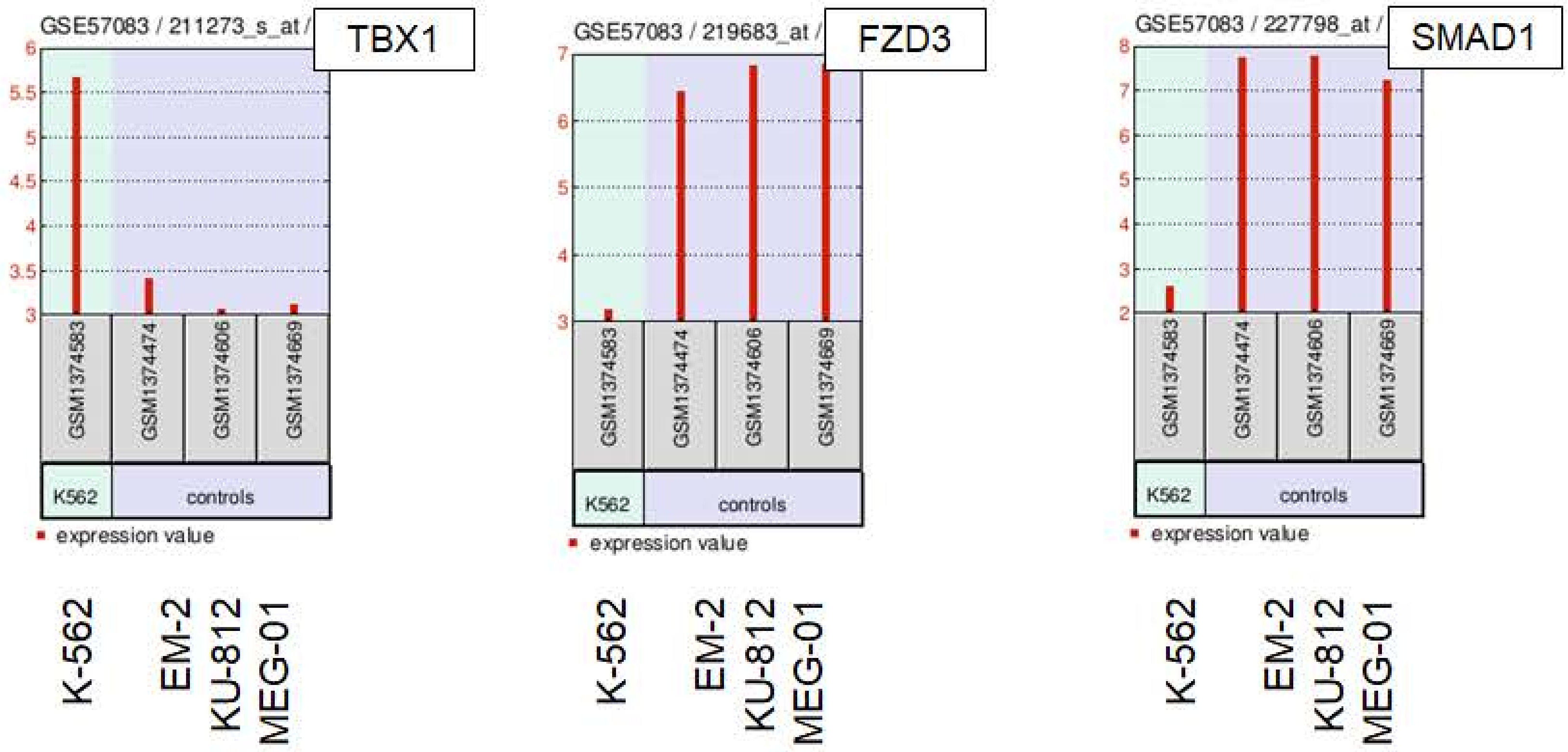

GEOR analysis of **CML patient** dataset GSE44589
comparing 10 top TBX1-high versus 10 TBX1-low controls prior Imatinib

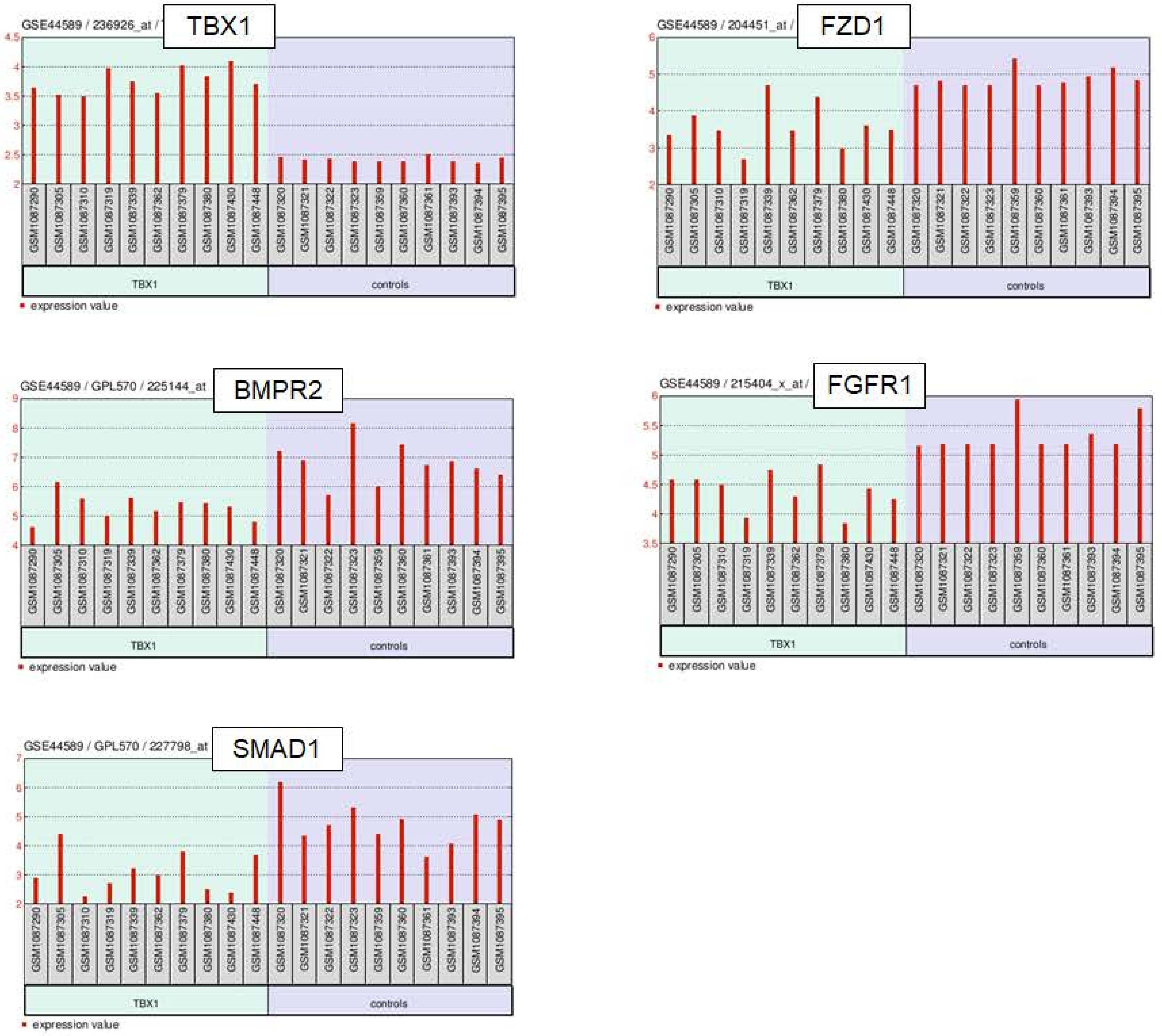

Figure S9: GEOR analysis of CML cell lines (dataset GSE57083) and patients (dataset GSE44589).

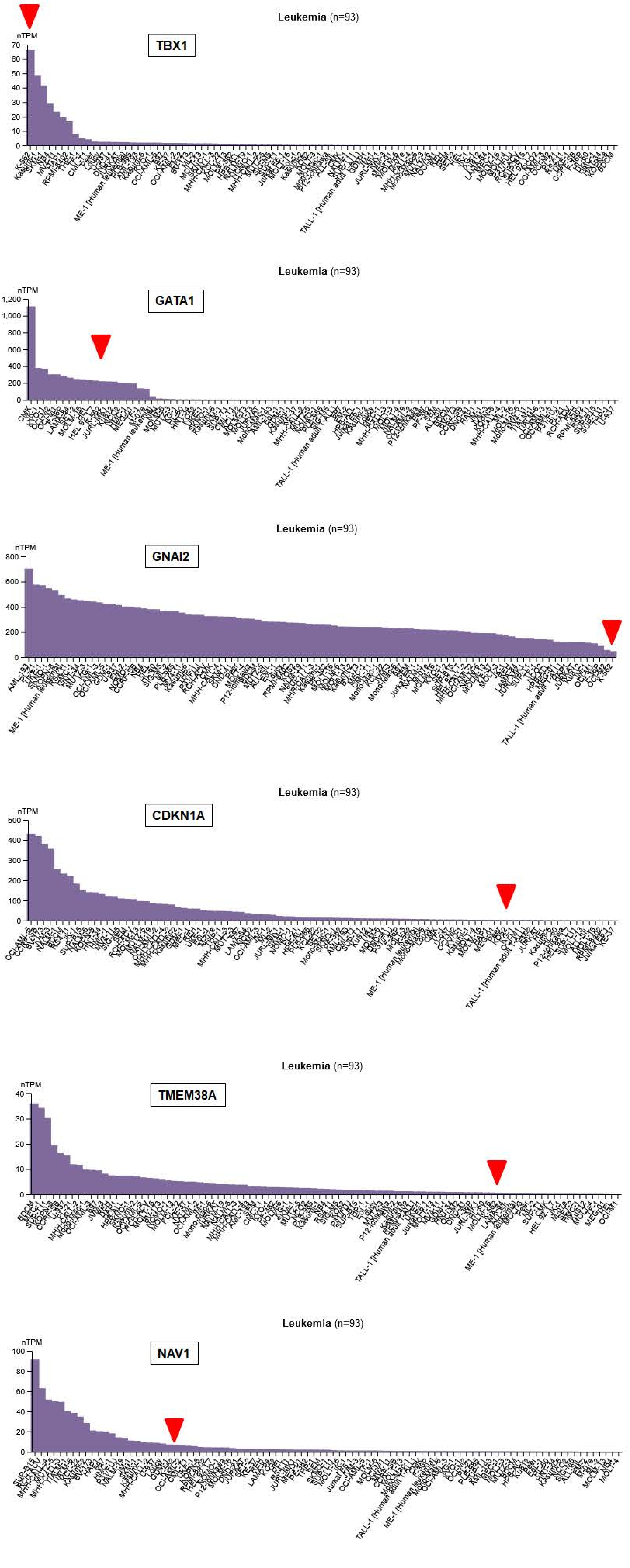

Figure S10: Selected gene activities in 93 leukemia cell lines using RNA-seq data from the Human Protein Atlas for TBX1, GATA1, GNAI2, CDKN1A, TMEM38A and NAV1.

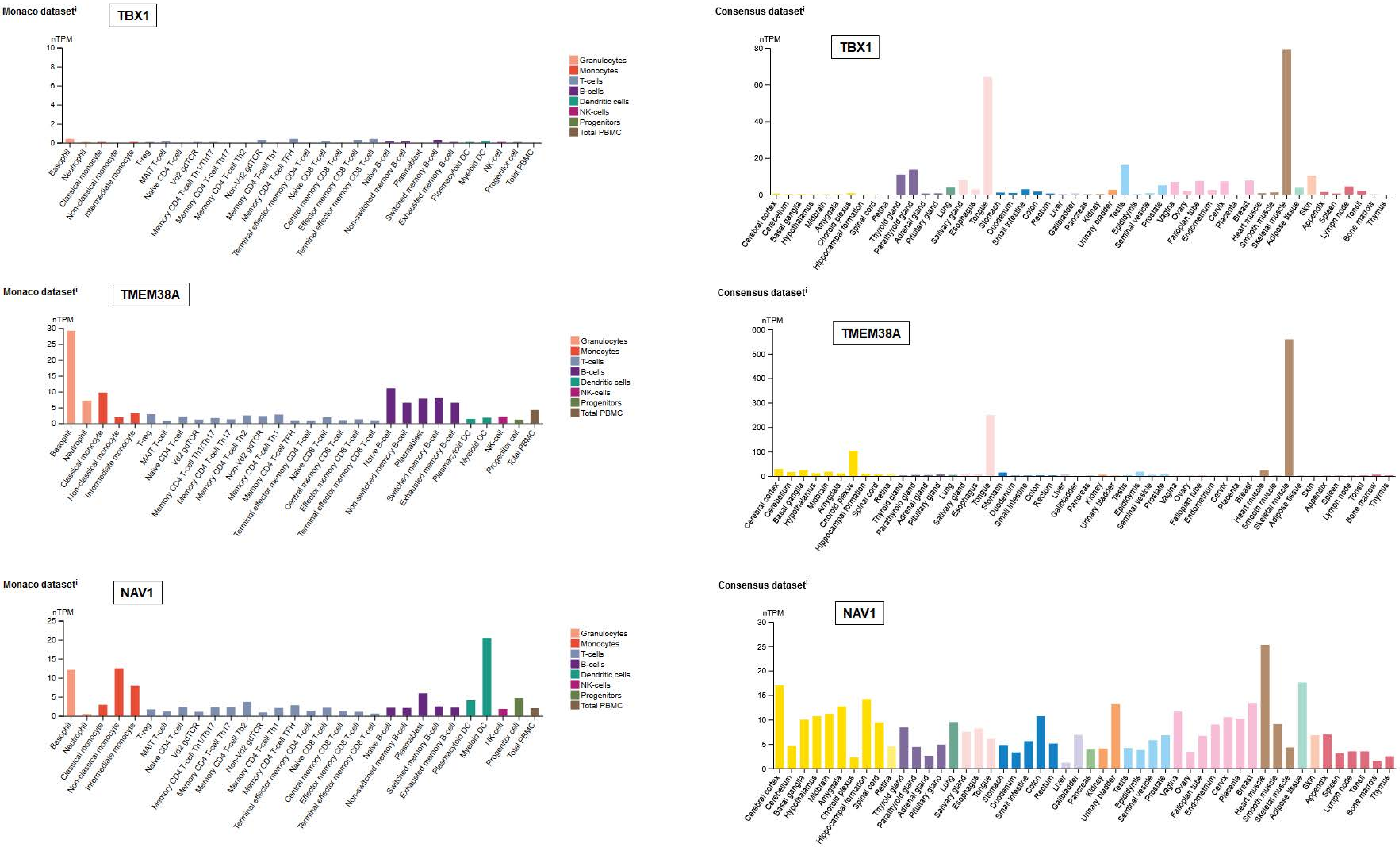

Figure S11: Gene activities for TBX1, TMEM38A and NAV1 using RNA-seq data from the Human Protein Atlas for hematopoietic cell types (Monaco dataset, left) and diverse tissue types (Consensus dataset, right).

external_gene_name	log2FoldChar	padj
AC093525.1	-6,49	9,38E-03
GPR157	-4,17	1,87E-89
AC008763.2	-3,90	9,99E-06
SULT1A4	-2,50	3,40E-05
SPATA17	-1,77	4,71E-02
AP000812.4	-1,74	2,62E-02
ACVR1C	-1,70	3,00E-02
INPP4B	-1,68	4,71E-02
DDX58	-1,28	3,19E-04
SMIM27	-1,13	1,42E-03
TANC2	-0,99	1,38E-02
P2RY11	-0,90	3,88E-02
ESPN	-0,88	2,53E-02
HSPA6	-0,87	4,36E-05
ZNF33B	-0,86	4,32E-02
SAMD11	-0,82	3,87E-03
SYT10	-0,79	3,49E-02
FADS3	-0,79	2,25E-14
LGALSL	-0,75	2,62E-02
HSPE1-MOB4	-0,72	2,57E-02
TBX1	-0,63	2,48E-10
CYRIA	-0,61	2,10E-03
PLAT	0,61	2,65E-02
GGT1	0,62	1,58E-02
GIPC3	0,63	8,99E-03
NAV1	0,68	1,43E-03
AL157935.2	0,92	1,78E-02
FAM114A1	0,97	1,17E-02
HERC3	1,21	5,17E-04
NOTCH3	1,95	3,40E-05
CAPN11	2,04	4,84E-02
TMEM38A	2,62	1,06E-07
DMBT1	3,48	3,72E-03
ZNF385B	5,44	4,71E-02

Table S1: List of potential TBX1 target genes. These genes were statistical significant differentially expressed in K-562 cells treated for TBX1-knockdown (performed in triplicate). Indicated are log2-fold change and the according p-value. The genes TBX1, TMEM38A and NAV1 are highlighted.
